# Case Report: Fetal growth restriction and prolonged gestation associated with umbilical cord torsion and entanglement in a Holstein dairy cow

**DOI:** 10.3389/fvets.2025.1704892

**Published:** 2025-12-15

**Authors:** Koto Ohsaki, Yuto Sano, Takashi Murakami, Yuki Ishiguro, Ayaka Ohtake, Ayano Sato, Ken Nakada, Tomochika Sugiura

**Affiliations:** 1 Laboratory of Theriogenology, Farm Animal Clinical Sciences, Department of Veterinary Medicine, School of Veterinary Medicine, Ebetsu, Hokkaido, Japan; 2 Laboratory of Veterinary Pathology, Pathobiology, Department of Veterinary Medicine, School of Veterinary Medicine, Ebetsu, Hokkaido, Japan; 3 Laboratory of Farm Animal Surgery, Farm Animal Clinical Sciences, Department of Veterinary Medicine, School of Veterinary Medicine, Ebetsu, Hokkaido, Japan

**Keywords:** bovine fetus, umbilical cord torsion, umbilical cord entanglement, fetal growth restriction, maternal serum analysis, parturition

## Abstract

This case report describes a rare instance of a bovine fetus with concurrent umbilical cord torsion and entanglement, which resulted in fetal growth restriction (FGR) and prolonged gestation. A 36-month-old primiparous Holstein cow, 285 days pregnant, was examined after failing to show signs of parturition. Fetal heartbeat was confirmed via abdominal ultrasonography until 295 days post-artificial insemination (AI) but was not detected thereafter, leading to a presumptive diagnosis of fetal death at 313 days post-AI. Following induction, the dead fetus was delivered at 316 days post-AI. Despite a gestational age of approximately 10.5 months, the fetus exhibited severe growth restriction that is equivalent to that of a normal 7-month-old fetus. The umbilical cord measured 40 cm—abnormally long for a 7-month-old fetus—and was tightly wrapped around the right hind limb with more than 360 degrees of torsion along its long axis. Maternal serum analysis revealed persistently high progesterone and markedly low estradiol concentrations before parturition, suggesting that the physiological process of parturition had not been initiated. It is speculated that the FGR resulted from chronic blood flow obstruction likely associated with the umbilical cord abnormalities, which were hypothesized to have occurred during the second trimester (approximately 4 months). The consequent absence of normal fetal signals to initiate parturition and the lack of periparturient endocrine changes contributed to prolonged gestation and underdeveloped mammary glands in the dam. This report represents the first detailed description of intrauterine umbilical cord abnormalities in cattle, demonstrating their potential to cause fetal developmental delay, prolonged gestation, and impaired mammary gland development.

## Introduction

The normal gestation period of Holstein dairy cows is approximately 280 days, and both calving difficulty and stillbirth rates increase when gestation extends beyond this duration (1, 2). Prolonged gestation period is relatively common in dairy cows, and could be caused by several factors such as month of conception, days in lactation, age of the dam, parity, fetal sex, and the presence of twins (2, 3).

In humans (4) and horses (5, 6), the umbilical cord typically coils along its long axis as a physiological phenomenon. However, little is known about the morphology of the bovine fetal umbilical cord in utero. Previous reports have shown that the umbilical cord length of bovine fetuses (*Bos Indicus*) is approximately 25.6 ± 1.3 cm around 150 days of gestation, and thereafter, the cord length remains constant without significant elongation (7). Furthermore, based on our observations of fetuses in the uterus of pregnant cattle carcasses and pathological findings of bovine pregnancies, the cord is either uncoiled or exhibits only a slight coiling of approximately 90 degrees. In horses, excessive coiling results from fetal rotation within the uterus, and it has been associated with abortion during the second trimester (8–11). Cord length has been identified as one of the factors contributing to overcoiling (8, 9, 12). Although umbilical cord abnormalities account for 46.2%−60.5% of all abortions in horses (8, 9), reports on umbilical cord abnormalities in bovine fetuses are extremely rare.

Umbilical cord entanglement occurs when the cord becomes tangled or wrapped around a part of the body of the fetus (13). In humans, umbilical cord entanglement around the neck (nuchal cord) is common, with an incidence rate of approximately 30% (14). The incidence of fetal umbilical cord entanglement is 14.7%−33.7% of all parturitions (15–17), and there are reports that entanglement around the body increases the risk of a low Apgar score (neonatal life evaluation) and low umbilical cord arterial pH in humans (18). In horses, cord entanglement is uncommon but has been reported in a few cases (8, 10, 19). In contrast, reports on the intrauterine morphology of the bovine umbilical cord are extremely limited, including a pathological survey in which torsion was identified in 1 of 180 aborted fetuses (0.6%) (20) and a brief report of a case of umbilical cord torsion as the cause of fetal mummification (21).

In the present study, we report a Holstein dairy cow, in which the umbilical cord torsion and entanglement were suspected to have occurred simultaneously in the fetus, leading to severe fetal growth restriction (FGR) and subsequent prolonged gestation. To our knowledge, this is the first detailed description of a bovine fetus with umbilical cord abnormality, including an estimation of the timing of onset.

## Case description

This case involved a 36-month-old primiparous Holstein dairy cow, which was 285 days pregnant at the initial examination (Table 1). Artificial insemination (AI) had been performed once on January 15, 2024 using Holstein semen. According to the owner, there were no signs of parturition even after the expected calving date, and udder development was poor. Rectal palpation and transrectal ultrasonography were performed by a veterinarian.

**Table 1:** Time line of the case.

**Timepoint**	**Date**	**Days of pregnant**	**Event**
	January 15, 2024	0	Artificial insemination (Holstein semen)
⋮	⋮	⋮	
Day 1	October 26, 2024	285	No abnormalities were detected with pregnant uterus
Day 10	November 5, 2024	295	The presence of a fetal heartbeat was confirmed via abdominal ultrasonography
Day 16	November 11, 2024	301	The fetal heartbeat could not be clearly detected via abdominal ultrasonography
Day 27	November 22, 2024	312	The fetal heartbeat could not be clearly detected via abdominal ultrasonography
Day 28	November 23, 2024	313	Placentome regression and irregular amniotic sac morphology were detected via transrectal ultrasonography Treated with 10 mg of dexamethasone
Day 29	November 24, 2024	314	Treated with 25 mg of prostaglandin F_2α_
Day 31	November 26, 2024	316	The fetus was delivered, and a pathological autopsy was performed

**Figure 1:**
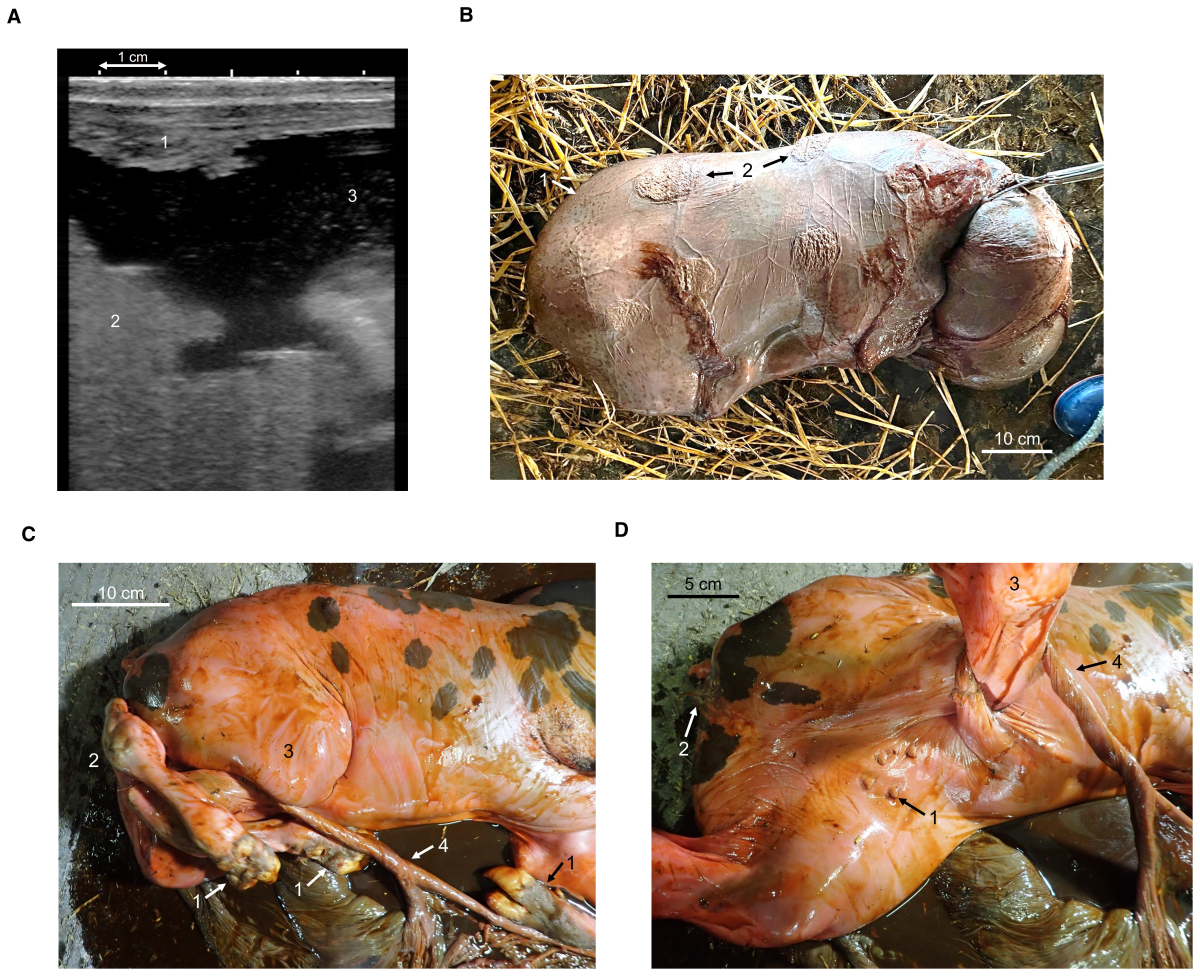
Transrectal ultrasonography image on Day 28. **(A)** Regression of placentomes (1), irregular amniotic sac (2), and floating debris in the allantoic fluid (3) are observed. Delivered fetus enveloped in the fetal membranes. **(B)** The fetal head on the right; chorion (1) and regressed cotyledons (2) are visible. Umbilical cord entanglement around the hind limb after removal of the membranes. **(C)** Hair extending from the coronary band to the fetlock (1), left hind limb (2), right hind limb (3), and entangled umbilical cord with torsion along its long axis (4). Close-up of the umbilical cord at the right hind limb. **(D)** The umbilical cord shows clear torsion along its longitudinal axis and is tightly wrapped around the right hind limb. Mammary buds (1), vulva (2), right hind limb (3), and entangled umbilical cord with torsion along its long axis (4).

On the first visit (Day 1; October 26, 2024; 285 days post-AI), the fetus was palpable on rectal palpation. The uterine artery was slightly thin, approximately 1.5 cm, but a sand-like pulsation (fremitus) was detected. Ultrasonography showed sufficient allantoic fluid and no placentome abnormalities, suggesting that the pregnancy was still progressing normally; therefore, a conservative approach was adopted. On Day 10 (November 5, 2024; 295 days post-AI), abdominal ultrasonography, performed following the method of Aziz (22), confirmed the presence of a fetal heartbeat from the right lower abdomen. Abdominal ultrasonography was also performed on Day 16 (November 11, 2024; 301 days post-AI) and Day 27 (November 22, 2024; 312 days post-AI). Although fetal ribs were visualized near the abdominal midline of the dam, the heartbeat could not be clearly detected. On Day 28 (November 23, 2024; 313 days post-AI), transrectal ultrasonography revealed placentome regression, irregular amniotic sac morphology, and floating debris in the allantoic fluid, leading to a diagnosis of fetal death (Figure 1A). On the same day, parturition was induced with a subcutaneous injection of 10 mg of dexamethasone (Aqueous-Dexamethasone-Injection A; ZENOAQ, Fukushima, Japan). The following day (Day 29; November 24, 2024; 314 days post-AI), 25 mg of prostaglandin F_2α_ (dinoprost tromethamine, Pronalgon EZ; Zoetis, Tokyo, Japan) was administered intramuscularly. On Day 31 (November 26, 2024; 316 days post-AI), the fetus was delivered, and a pathological autopsy was performed.

The delivered dead fetus was enclosed in the chorioallantoic membrane, on the surface of which cotyledons that had been completely detached and regressed from the maternal placenta were observed (Figure 1B). The placental tissue showed advanced autolysis, with very little allantoic or amniotic fluid present and the fetus exhibiting generalized hydrops. Body length of the fetus was 72 cm and weight was 20.0 kg. The fetus had clearly defined mammary buds and a vulva, indicating that it had already differentiated into a female. The eyelids of the fetus were separate and had eyelashes. No internal organ abnormalities were identified, but the fetus was markedly underdeveloped. Hair growth was present on the inner side of the fetal pinnae, on the muzzle, and from the coronary band to the fetlock.

The umbilical cord extended caudally between the hind limbs, then turned cranially below the right knee, wrapping tightly around the right hind limb before becoming entangled along the path to the placenta. The cord itself also showed more than 360 degrees of clockwise torsion along its long axis, with marked tension and compression from the right hind limb (Figures 1C, D). In addition, the left hind limb crossed over the hock of the right hind limb (Figure 1C).

Upon autopsy, the umbilical cord, which had been entangled around the right hind limb, was untangled and relaxed, measuring 40 cm in length and 1.7 cm in diameter at the base of the cord, with no evidence of crushed areas. Although joint loosening was present due to postmortem changes, pathological examination revealed no morphological abnormalities in either hind limb. The hypothalamus, pituitary gland, and other endocrine organs exhibited advanced autolysis, making it impossible to determine the presence of potential abnormalities. Considering the postmortem changes, no other morphological abnormalities that could have explained the cause of fetal death were identified.

**Figure 2:**
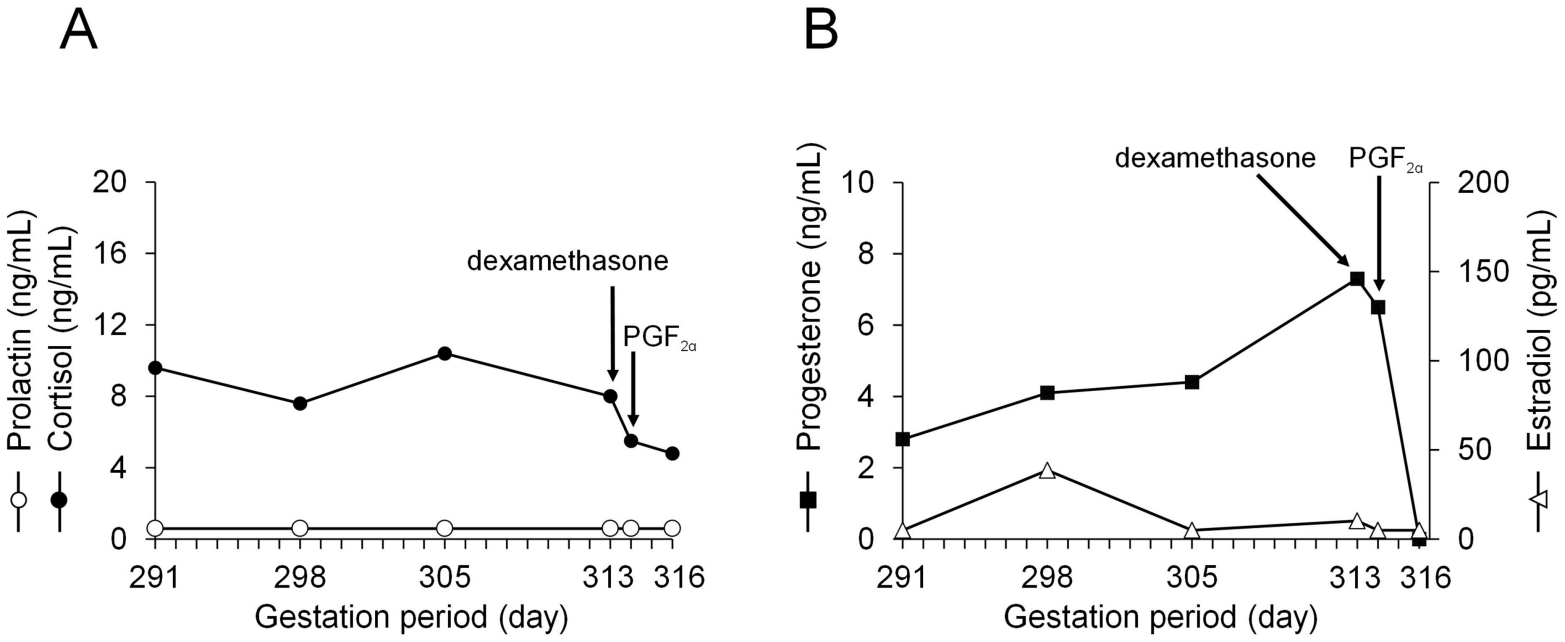
Maternal serum analysis. Concentrations of prolactin and cortisol **(A)**, and concentrations of progesterone and estradiol **(B)**. PGF_2α_: prostaglandin F_2α_.

The general condition of the cow remained good from Day 1 until calving, and milk ejection was observed after calving. Blood samples were collected on Days 6, 13, 17, 18, and 19 from the coccygeal vein for hormone analysis. After centrifugation at 1,700 × *g* for 15 min, serum was obtained, and concentrations of cortisol (Cortisol EIA Kit; Arbor Assays, Michigan, USA), progesterone, estradiol, and prolactin were measured (Kishimoto First Clinical Laboratory Center, Hokkaido, Japan). Cortisol concentrations remained slightly elevated throughout the sampling period, whereas prolactin concentrations were consistently low (Figure 2A). Progesterone concentrations remained at a normal high level, whereas estradiol concentrations remained at a very low level (Figure 2B). Following dexamethasone and prostaglandin F_2α_ administration, progesterone concentrations decreased sharply. In this case, milk yields of 26.0, 31.8, and 32.5 kg were recorded on Days 16, 51, and 81, respectively, after induced parturition. Although the udder was severely shrunken before calving and lactation was not anticipated, gradual udder swelling was observed after the induced parturition procedure, and lactation proceeded normally.

## Discussion

**Figure 3:**
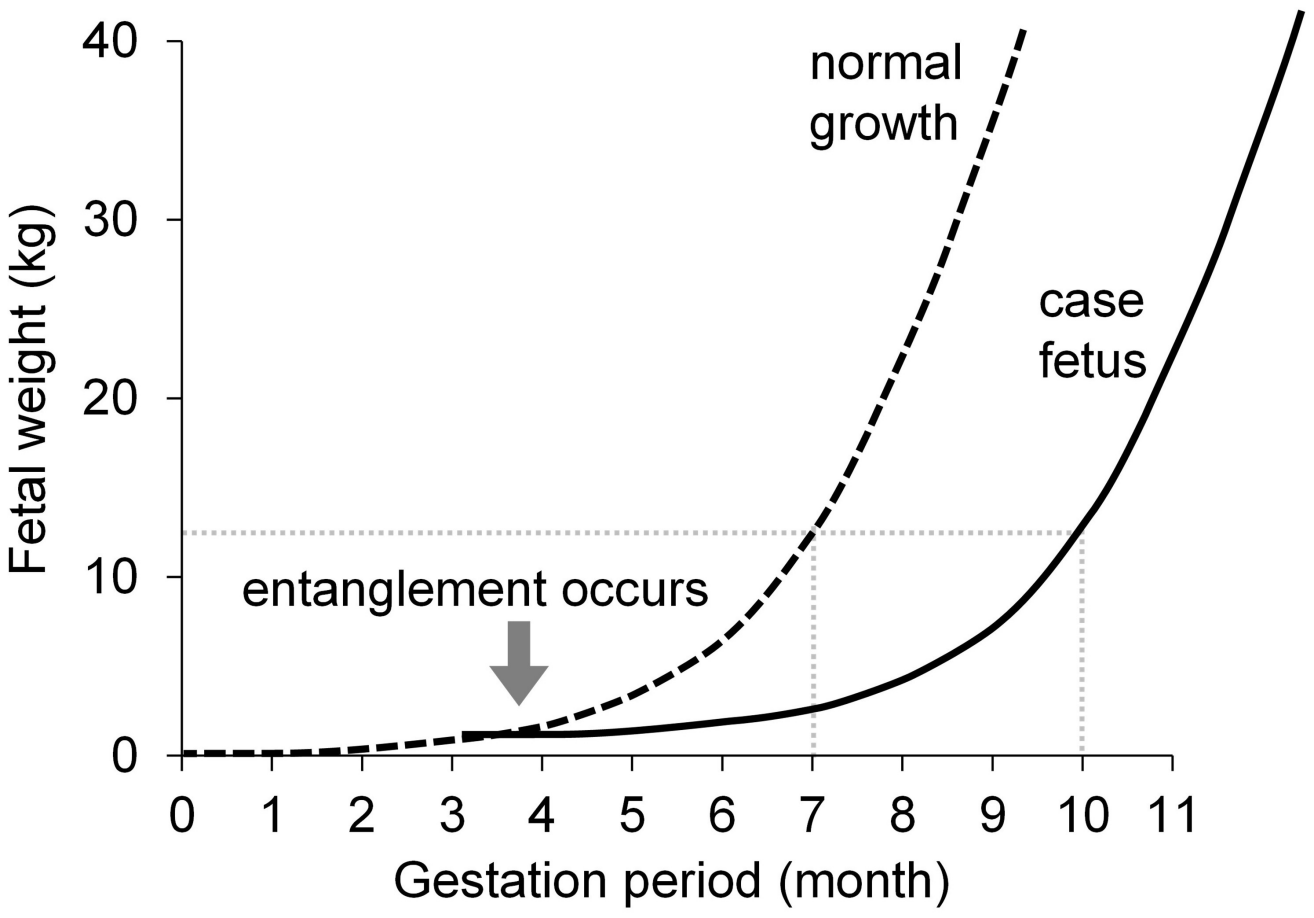
Presumptive fetal growth curve. Dashed line: normal fetal growth curve (25); solid line: presumed growth curve of the case fetus. The fetus in this case had a developmental delay equivalent to that of a normal 7-month-old fetus at 10 months of gestation.

The fetus in this case, delivered at approximately 10.5 months of gestation, showed hair growth on the inner pinnae, from the coronary band to the fetlock, and on the muzzle, with a body length of approximately 72 cm. Based on these developmental characteristics, the fetus was at a developmental stage that is equivalent to that of a normal 7-month-old fetus (23). Therefore, at the time of the last confirmed fetal heartbeat on Day 10 (295 days of gestation, approximately 10 months old), a developmental delay of approximately 3 months was presumed. According to a report by Hammond (24), the weights of a normal 7-month-old bovine fetus and its associated fetal fluids are approximately 12.5 and 7.5 kg, respectively. The fetus in this case weighed 20.0 kg at delivery, and since it was a hydrops fetus that had absorbed nearly all fetal fluids, its estimated weight before the onset of hydrops would have been 12.5 kg, with the assumption that 7.5 kg of fetal fluids were absorbed. This estimated weight aligns with the fetal growth levels reported by Krog et al. (23) and Hammond (24). By shifting the fetal growth curve from Bauman and Currie (25) to the right and plotting the growth curve for the fetus in this case, which corresponded to a 7-month growth stage at 10 months of gestation, a presumptive growth curve, such as that shown in Figure 3, was obtained. As shown in Figure 3, the divergence point between normal growth and that of the fetus in this case appears to be approximately 4 months of gestation, suggesting that the umbilical cord torsion and entanglement were hypothesized to have occurred at that stage, leading to subsequent developmental delay.

Abnormal umbilical cord length has been reported as a major risk factor for over coiling or entanglement in humans (26, 27) and horses (8, 9, 12). The normal umbilical cord length of a newborn calf is 30–40 cm (28). In this case, the umbilical cord measured 40 cm, despite the fetus corresponding to a developmental stage of approximately 7 months, when the normal cord length is approximately 30 cm (29). Thus, the cord of this fetus was more than 10 cm longer than that of a normal fetus of equivalent body length. This excessive length may have contributed to increasing the freedom of movement of the fetus within the amniotic cavity. During the second trimester (approximately 4 months), when amniotic space is ample, the fetus likely moved actively, and this, combined with the long umbilical cord, accidentally resulted in both torsion due to fetal rotation and entanglement around the right hind limb. As no abnormalities were detected in either hind limb, the entanglement was not attributed to functional impairment. Reported factors contributing to umbilical cord elongation include uterine space and tension from fetal movement in humans (30), parity in humans (31), and genetic predisposition in horses (9). The cause of the excessively long umbilical cord in this case remains unclear; however, given the rarity of torsion or entanglement reports in bovine fetuses, the elongation may have been idiopathic. In this case, the severe autolysis of the fetus posed challenges for performing a histopathological examination and eliminating the possibility of infection. However, fetal infection, metabolic abnormalities, and genetic factors are also possible causes of significant FGR.

In humans, both excessive coiling and entanglement of the umbilical cord have been associated with FGR (32, 33). Even mild nuchal cord has been linked to nutritional or hypoxic stress (14). An ovine fetus study demonstrated that 50% compression of the umbilical cord reduced blood flow to the lungs and liver and decreased oxygen delivery to the peripheral tissues (34). Therefore, it is speculated that the fetus in this case suffered from FGR due to reduced umbilical blood supply caused by the umbilical cord torsion and entanglement around the right hind limb. Although not immediately fatal, this restriction likely impaired development over time.

On Day 10, when the fetal heartbeat was last confirmed, the fetus was located in the right lower abdomen. However, in subsequent ultrasonography, the fetal ribs were visualized near the abdominal midline of the dam. This positional change may have increased the tension on the umbilical cord, which in turn increased the compression of the vessels (umbilical vein and arteries). The resulting reduction in blood flow likely contributed to fetal death. Since bovine fetuses undergo rapid growth in late gestation, restricted umbilical blood supply may have further exacerbated the inability to meet the high nutritional and oxygen demands at that stage, ultimately leading to fetal demise.

In cattle, the normal mechanism of parturition involves increased fetal cortisol, which activates 17α-hydroxylase in the placenta. This, in turn, elevates estrogen and decreases progesterone concentrations in the maternal circulation (35). Specifically, blood estrogen concentrations begin to increase around 250 days of gestation, peaking at approximately 8 ng/ml 2–5 days before parturition (36). Blood progesterone concentrations range from 3–10 ng/ml during the third trimester of pregnancy and then gradually decline approximately 3 weeks before parturition (36, 37). Blood cortisol concentrations range from 3–5 ng/ml during the third trimester of pregnancy, then rapidly increase approximately 1 week before parturition, reaching approximately 10 ng/ml on the day of parturition (36, 37). Blood prolactin concentrations also rapidly increase approximately 2 weeks before parturition, reaching approximately 200 ng/ml on the day of parturition (36). The resulting increase in the estrogen/progesterone ratio stimulates endometrial prostaglandin F_2α_ production, initiating uterine contractions (labor pains).

In the present case, cortisol concentrations in the cow remained at a slightly elevated level (Figure 2A). This is believed to be an effect of the fetal cortisol concentration, in response to reduced umbilical blood supply due to umbilical cord torsion and entanglement. The animal did not show any symptoms such as fever or loss of appetite during the observation period and remained consistently healthy in an environment with adequate cow comfort. Although progesterone remained at a high level, estradiol concentrations remained at a very low level (Figure 2B), keeping the estrogen/progesterone ratio consistently low. This imbalance was not high enough to activate placental 17α-hydroxylase, which explains why spontaneous parturition (abortion) did not occur (Figure 2B).

Prolactin promotes mammary cell proliferation and milk production (38). The persistently low concentrations of prolactin and estradiol likely explain the poor mammary gland development and shriveled udders in the cow before calving. Progesterone also suppresses lactose synthesis by inhibiting α-lactalbumin synthesis, thereby inhibiting milk secretion (39). In this case, progesterone concentrations decreased sharply following prostaglandin administration, which induced parturition. This decline likely enabled mammary gland development and the onset of milk ejection after calving. Furthermore, the continued function of the placenta until 295 days of gestation may have supported mammary development postpartum. Although initial milk production immediately after calving was low, daily milking stimulation contributed to progressive mammary development and subsequent increases in milk yield.

## Conclusion

Severe fetal growth restriction (FGR) in this case was attributed to umbilical cord torsion and entanglement, which were hypothesized to have occurred in a bovine fetus at approximately 4 months of gestation. Umbilical cord abnormalities in bovine fetuses are extremely rare; however, this study showed that such abnormalities can lead to prolonged gestation and impaired mammary gland development in the dam. Limited reports on necropsy documentation and imaging under field conditions may also contribute to the underestimation of the prevalence of umbilical cord abnormalities in bovine fetuses.

## Data Availability

The original contributions presented in the study are included in the article/Supplementary material, further inquiries can be directed to the corresponding author.

## Supplementary material

Supplementary Video S1 Fetal heartbeat video from maternal right lower abdominal ultrasonography on Day 10 (November 5, 2024; 295 days post-AI).

Supplementary Video S2 The video of fetal membrane incision.

Supplementary Figure S1 The fetal umbilical cord with only a slight coiling at approximately 3 months of gestation.
