# Supplementary figures and images for: Case Report: Fetal growth restriction and prolonged gestation associated with umbilical cord torsion and entanglement in a Holstein dairy cow
Source: Front Vet Sci. 2025 Dec 15;12:1704892. doi: 10.3389/fvets.2025.1704892 (PMC12745216; doi:10.3389/fvets.2025.1704892)

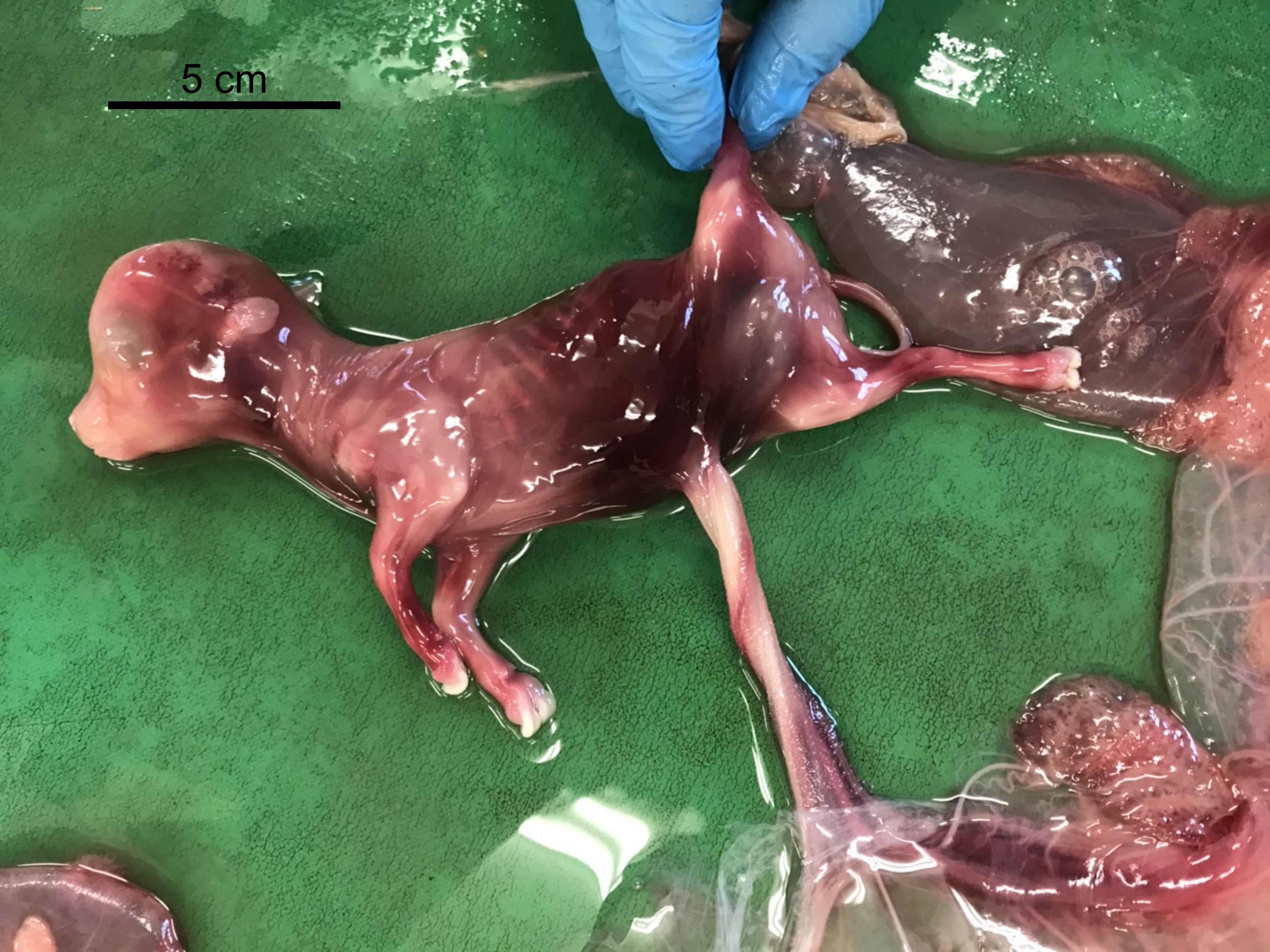

Supplement: Supplementary Figure S1 — The fetal umbilical cord with only a slight coiling at approximately 3 months of gestation. [file Image_1.JPEG]
